# Characterization of Virulence Factors in *Candida* Species Causing Candidemia in a Tertiary Care Hospital in Bangkok, Thailand

**DOI:** 10.3390/jof9030353

**Published:** 2023-03-14

**Authors:** Natnaree Saiprom, Thanwa Wongsuk, Worrapoj Oonanant, Passanesh Sukphopetch, Narisara Chantratita, Siriphan Boonsilp

**Affiliations:** 1 Department of Microbiology and Immunology, Faculty of Tropical Medicine, Mahidol University, Bangkok 10400, Thailand; natnaree.sai@mahidol.ac.th (N.S.); natthanej.lup@mahidol.ac.th (P.S.); narisara@tropmedres.ac (N.C.); 2 Department of Clinical Pathology, Faculty of Medicine Vajira Hospital, Navamindradhiraj University, Bangkok 10300, Thailand; thanwa@nmu.ac.th; 3 Department of Basic Medical Science, Faculty of Medicine Vajira Hospital, Navamindradhiraj University, Bangkok 10300, Thailand; worrapoj@nmu.ac.th; 4 Mahidol-Oxford Tropical Medicine Research Unit, Faculty of Tropical Medicine, Mahidol University, Bangkok 10400, Thailand

**Keywords:** *Candida* spp., candidemia, virulence factors, *ERG11* mutation

## Abstract

Candidemia is often associated with high mortality, and *Candida albicans, Candida tropicalis, Candida glabrata,* and *Candida parapsilosis* are common causes of this disease. The pathogenicity characteristics of specific *Candida* spp. that cause candidemia in Thailand are poorly understood. This study aimed to characterize the virulence factors of *Candida* spp. Thirty-eight isolates of different *Candida* species from blood cultures were evaluated for their virulence properties, including exoenzyme and biofilm production, cell surface hydrophobicity, tissue invasion, epithelial cell damage, morphogenesis, and phagocytosis resistance; the identity and frequency of mutations in *ERG11* contributing to azole-resistance were also determined. *C. albicans* had the highest epithelial cell invasion rate and phospholipase activity, with true hyphae formation, whereas *C. tropicalis* produced the most biofilm, hydrophobicity, protease activity, and host cell damage and true hyphae formation. *ERG11* mutations Y132F and S154F were observed in all azole-resistant *C. tropicalis*. *C. glabrata* had the most hemolytic activity while cell invasion was low with no morphologic transition. *C. glabrata* was more easily phagocytosed than other species. *C. parapsilosis* generated pseudohyphae but not hyphae and did not exhibit any trends in exoenzyme production. This knowledge will be crucial for understanding the pathogenicity of *Candida* spp. and will help to explore antivirulence-based treatment.

## 1. Introduction

Candidemia caused by blood-borne *Candida* is the most common fungal bloodstream infection (BSI) in hospitalized patients [1] and has a high mortality rate of almost 50% [2]. *Candida albicans* is the main cause of candidemia globally, but the incidence of non-*C. albicans Candida* (NAC) species such as *Candida glabrata*, *Candida tropicalis*, and *Candida parapsilosis* has also increased [3,4]. These four species, *C. albicans* (42.1%), *C. glabrata* (26.7%), *C. parapsilosis* (15.9%), and *C. tropicalis* (8.7%) are the most common causes of invasive candidemia and account for approximately 90% of all *Candida* BSIs [5]. In Thailand, *C. tropicalis* was the most frequent species isolated from blood samples [6,7]. The NAC spp. represent a therapeutic challenge given the different antifungal susceptibility profiles of different *Candida* species [1]. The azole antifungal drugs are most commonly used to fight infections caused by *Candida* spp., and long-term fluconazole treatments for chronic infections have enabled *Candida* spp. to develop resistance to these, with *C. tropicalis*, *C. glabrata*, and *C. krusei* frequently resistant to azole antifungals [8,9]. Various azole-resistance mechanisms in *Candida* species have been described, including overexpression of cytochrome P450 lanosterol 14alpha-demethylase (Erg11) and different drug efflux transporters (CDR and MDR) [10,11,12]. In addition, the mutation in the *ERG11* gene reduces azole affinity for the target enzyme CYP51A1 and is one of the mechanisms contributing to azole resistance in clinical isolates. Greater understanding of specific point mutations in *ERG11* linked to azole resistance could help identify resistant strains, adjust treatment strategies, and rationally design new drugs.

The propensity for *Candida* species to cause disseminated invasive infections may be linked to potential virulence factors [13]. *C. albicans*, *C. tropicalis*, and *C. glabrata* are the more virulent species, and infections with them are more likely to result in death [14]. *Candida* pathogenicity is facilitated by various virulence factors that enable adherence and invasion to the target cell surface, formation of biomass, yeast-to-hyphae transition, production of tissue-damaging hydrolytic enzymes (e.g., proteases, phospholipases, and hemolysins), and evasion of immune cells [15,16,17]. Virulence factors can differ depending on the infecting species, geographical origin, infection type, infection site, and host reaction. Previously, we discovered that more than half of the genotyped isolates causing BSIs in patients in our hospital had new genotypes as defined using multilocus sequence typing (MLST) [6]. These novel genotypes may result in different virulence phenotypes for isolates from Thailand. Despite extensive research into identifying pathogenic factors in *Candida* species, particularly *C. albicans*, little is known about virulence factors of the current *Candida* species causing BSIs in Thailand. Knowledge of these virulence factors is crucial for understanding the pathogenesis of candidemia and can help identify new targets for antivirulence-based therapeutics to treat infecting *Candida* spp. Therefore, this study aimed to characterize the virulence factors, including extracellular enzymatic activities, cell surface hydrophobicity (CSH), and biofilm formation, host-pathogen interaction, and the mutation pattern of *ERG11* genes in *Candida* species isolated from patients with candidemia in Bangkok, Thailand.

## 2. Materials and Methods

### 2.1. Yeast Isolates and Ethical Statement

The 38 clinical isolates of *C. albicans*, *C. glabrata*, *C. parapsilosis* and *C. tropicalis* were obtained in our previous study and were isolated from patients with candidemia between June 2018, and July 2019, at the Department of Central Laboratory and Blood Bank, Faculty of Medicine, Vajira Hospital, Navamindradhiraj University, Bangkok, Thailand. Isolate information is listed in Table S1. Briefly, isolates were identified using matrix-assisted laser desorption/ionization time-of-flight mass spectrometry (Bruker Microflex, Bremen, Germany) and internal transcribed spacer sequence. Antifungal susceptibility testing was evaluated using Sensititre YeastOne^TM^ (Trek Diagnostic Systems, Cleveland, OH, USA) [6]. *Candida* yeast cells were cultured on Sabouraud dextrose agar (SDA) (Oxoid, Cambridge, UK) and incubated overnight at 30 °C. A single colony was inoculated into Sabouraud dextrose broth (SDB) (Oxoid, Cambridge, UK) and incubated at 30 °C for 24 h for virulence factor assessment.

This work was approved by The Ethics Committee of the Faculty of Medicine Vajira Hospital, Navamindradhiraj University (COA 072/2565, Study code 315/64E) and Institutional Biosafety Committee, Faculty of Tropical Medicine, Mahidol University (FTM-IBC-22-01).

### 2.2. Hydrolytic Enzyme Activity Assays

Extracellular protease activity was tested by inoculating *Candida* isolates on bovine serum albumin (BSA) agar with some modifications from previous studies [18,19]. The BSA agar contained 0.1% KH_2_PO_4_, 0.05% MgSO_4_, 2% agar, 0.01% yeast extract, and 0.2% BSA adjusted to pH 4.5. A suspension (5 μL of 10^6^ cells/mL) of each strain was inoculated onto BSA medium, and plates were incubated at 37 °C for up to 5 days. A white precipitation zone around the colonies represented the cleavage of BSA and positive protease activity. The diameters of the yeast colony and the white precipitation zone were recorded.

To evaluate hemolytic activity, *Candida* yeast cells were adjusted to an inoculum size of 1 × 10^6^ cells/mL in SDB medium. The cell suspension (5 μL) was inoculated in duplicate on the surface of SDA supplemented with 3% glucose and 7% fresh sheep blood [20]. Plates were dried at room temperature and incubated for 48 h at 37 °C. After the incubation period, the presence of a translucent halo indicated positive hemolysis, and the diameters of the yeast colony and the clear zone were measured.

*Candida* phospholipase activity was tested on egg yolk medium containing 13.0 g/L SDA, 11.7 g/L NaCl, 1.48 g/L CaCl_2_.2H_2_O_2_, 2% agar, and 10% sterile egg yolk [19]. Yeast cells were grown overnight in SDB before adjustment to a McFarland standard of 0.5 (10^6^ cells/mL). A standard inoculum (5 μL) of the test strain was aseptically inoculated onto egg yolk agar. Plates were dried at room temperature and then incubated at 37 °C for 48 h. A white precipitation zone surrounding colonies was considered positive for extracellular phospholipase activity, and the diameters of the white precipitation zone and the yeast colony were measured.

The levels of phospholipase, proteinase and hemolytic activity were represented by the proteolytic zone (Pz value), which was the ratio of the diameter of the yeast colony to the overall diameter of the colony plus that of the white precipitation, clear halo, or translucent halo zones, respectively [21]. All hydrolytic enzyme activity experiments were performed in duplicate and repeated at least twice times. The isolate Pz values were established by measuring in duplicate the diameter of the colony and enzyme activity zone. The Pz values were averaged from two separate experiments, each carried out in duplicate.

### 2.3. CSH Assay

CSH was assessed using the microbial hydrocarbon adhesion assay [22]. Yeast cells were grown overnight in Sabouraud dextrose broth (SDB) at 37 °C and then harvested and washed twice with phosphate-buffered saline (PBS). A yeast cell suspension with an optical density at 600 nm (OD600) between 0.4 and 0.5 was prepared in PBS (A0). The yeast suspension (3 mL) was overlaid by 0.4 mL of the hydrophobic hydrocarbon n-hexadecane (Sigma-Aldrich, Burlington, MA, USA). Cells were vortexed for 2 min and incubated for 10 min at 30°C to allow phase separation. The aqueous phase was measured (A1) at OD600. The decrease in absorbance was used to calculate the percentage of hydrophobicity of the cell surface (% hydrophobicity): hydrophobicity (%) = [1 − (A1/A0)] × 100. CSH tests were performed in at least three independent experiments, each carried out in duplicate to obtain an average value of % CSH value.

### 2.4. Biofilm Formation

Yeast cells were grown overnight in SDB at 37 °C and then were adjusted to a 0.5 McFarland standard with SDB medium. Yeast suspension (100 μL) was seeded into wells in a 96-well flat bottom plate and incubated at 30 °C for 48 h. Wells were then washed twice with PBS to remove non-adhering cells. Empty wells were allowed to dry for 30 min, and then 200 μL of 0.4% crystal violet was added to each well and incubated for 45 min at room temperature. The plate was washed gently twice using distilled water, and 200 μL of absolute ethanol was added to destain the biofilm. The plate was then incubated for 45 min at room temperature. A 150 μL volume of eluted crystal violet was transferred to a new 96-well plate, and the OD at 590 nm was measured using Sunrise^TM^ ELISA reader (Tecan Group Ltd., Männedorf, Switzerland). Sterile SDB without yeast cells was used as negative control [23]. Each isolate was performed in two independent experiments and each experiment was repeated at least eight times. The biomass of each isolate was presented as the average OD value based on two independent experiments.

### 2.5. Candida spp. Invasion Assay

The invasion properties of different *Candida* species were analyzed using an invasion assay with adenocarcinomic human alveolar basal epithelial cell (A459 cell) monolayers in 24-well plates. A549 cells were cultured in cell culture flasks at 37 °C in 5% CO_2_ in RPMI 1640 medium (GibcoTM, New York, NY, USA) containing 10% fetal bovine serum (FBS) (HyClone^TM^, Pasching, Austria) until 80% confluence was achieved. The A549 cell monolayer was washed twice with 5 mL of Dulbecco’s phosphate-buffered saline (DPBS). Cells were dissociated using 1 mL of 0.25% Trypsin-EDTA solution (GibcoTM, New York, NY, USA) and incubated at 37 °C for 2 min. Trypsin was inactivated by adding RPMI 1640 medium containing 10% FBS. The cell suspension was centrifuged for 5 min at 123× *g*. The pellet was resuspended in 1 mL of RPMI 1640 medium containing 1% FBS. Cell number and cell viability were counted using 0.4% Trypan Blue staining. A549 cells were seeded at 1 × 10^5^ cells per well on sterile 12-mm diameter glass round coverslips placed in wells in a 24-well plate and incubated overnight. *Candida* yeast cells were suspended in RPMI containing 1% FBS to a 0.5 McFarland standard (approximately 1 × 10^6^ CFU/mL). A549 cells were infected with *Candida* species at a multiplication of infection (MOI) of 5 and incubated for 4 h at 37 °C in 5% CO_2_. An uninoculated cell line served as a negative control. After 4 h, cultures were washed twice with PBS to remove non-adhering yeast and fixed with absolute ethanol. *Candida* internalized into cells was observed using Gram’s staining. The percentage of invading yeast cells was determined by dividing the number of (partially) internalized cells by the total number of adherent cells and multiplying by 100 [24]. At least 100 yeast cells were counted. *Candida* cells with small daughter cells were regarded as one cell. Each isolate was performed in duplicates on at least two separate occasions. The percentage of invading yeast cell values was averaged from two separate experiments.

### 2.6. Cytotoxicity Assay

To determine the ability of *Candida* species to damage human epithelial cells, the release of lactate dehydrogenase (LDH) was determined after 18 h of infection at MOI of 5. A549 epithelial cells were cultured at 37 °C in 5% CO_2_ in RPMI containing 10% FBS until 80% confluence. A549 cells were seeded at 1.5 × 10^4^ cells per well in RPMI containing 1% FBS in 96-well plates and incubated overnight. *Candida* yeast cells grown overnight in SDB were washed three times, suspended in PBS, and adjusted to a 0.5 McFarland standard in RPMI plus 1% FBS. Yeast cell suspension (75 μL) in RPMI medium plus 1% FBS was transferred onto cultured A549 cells and incubated for 18 h at 37 °C in 5% CO_2_. Cell supernatants were collected after 18 h for LDH testing using the CytoTox-96 nonradioactive cytotoxicity assay (Promega, Madison, WI, USA). Initially, 50 μL of each control (RPMI, RPMI with lysis buffer, cell lysate, and A549 cell supernatant), and the coculture supernatant samples were added to a 96-well plate. Next, 50 μL of the CytoTox 96 reagent was added, and the plate was incubated for 30 min in the dark. Subsequently, 50 μL of a stop solution containing 1 M acetic acid was added to each well, and the absorbance was measured at 492 nm. Culture media were used as blanks for subtracting the culture medium background from all absorbance values. The percentage of the LDH release in the coculture medium was calculated according to the following formula:

% LDH release = (Experimental supernatant – A549 cell supernatant) × 100

(A549 cell lysate – A549 cell supernatant)

Cytotoxicity assays were performed at least twice in triplicate separate experiments. The cytotoxic activity of each isolate was presented as the average %LDH release derived from three independent experiments.

### 2.7. Fluorescent Staining and Confocal Microscopy

Strains VJR H2246, VJR H1584, VJR H0668, and VJR H0235 were selected as representative strains of *C. albicans*, *C. glabrata*, *C. tropicalis*, and *C. parapsilosis,* respectively, for describing the *Candida*–epithelial cell interaction. *Candida* spp. were cultured on SDA at 37 °C overnight. *Candida* spp. were resuspended in 2 mL of PBS and adjusted to a 0.5 McFarland standard solution. Fluorescent staining was performed as previously described with modifications [25]. Briefly, A549 cells suspended in RPMI containing 1% FBS were seeded at 5 × 10^5^ cells/well on a sterile glass coverslip placed in a 6-well tissue culture plate and incubated overnight at 37 °C with 5% CO_2_. The A549 cell monolayer was infected with *Candida* spp. At MOI of 5 for 1 or 4 h, after which cells were washed three times with PBS. Cells were fixed with 4% paraformaldehyde in PBS for 30 min at room temperature and permeabilized with 0.5% Triton X-100 (Sigma-Aldrich, Waltham, MA, USA) for 30 min at room temperature. After washing three times with PBS, yeast cells were stained by incubating with 10% Calcofluor white solution (Sigma-Aldrich, Markham, ON, Canada) in PBS for 15 min at 37 °C, then washing three times with PBS [26]. The actin cytoskeleton was stained with phalloidin conjugated with Alexa Fluor 647 (1:1000; Invitrogen, Buffalo, NY, USA) by incubation at 37 °C for 1 h. Stained cells were washed three times with PBS, and then the coverslips were mounted on glass slides using 8 μL of ProLong Gold antifade reagent (Invitrogen, NY, USA). Confocal microscopy was performed with a laser scanning confocal microscope (LSM 700; Carl Zeiss, Germany) using a 100× objective lens with oil-immersion and Zen software (2010 edition, Zeiss, Germany). The excitation and emission wavelengths were 352/461 for Calcofluor white and 594/633 for Alexa Fluor 647. Each isolate of the representative strains was performed in duplicate on at least two separate occasions. The confocal microscopy images were taken from two independent experiments.

### 2.8. Human Monocyte Uptake of Candida Cells

*Candida* spp. were cultured on SDA at 37 °C overnight and were then resuspended with 2 mL of PBS and adjusted to a McFarland standard solution of 3 (approximately 1.5 × 10^8^ CFU/mL). A total of 1 × 10^6^ cells were stained with 10 µg/mL carboxyfluorescein succinimidyl ester (CFSE) (BD company, Franklin Lakes, NJ, USA) and incubated for 30 min at 37 °C. *Candida* spp. was washed three times with 200 µL of Dulbecco’s PBS (DPBS) (HyClone^TM^, Pasching, Austria), followed by centrifugation for 5 min at 1109× *g*; the pellet was resuspended with 20 µL DPBS.

THP-1 (ATCC TIB-22) human monocyte line in RPMI 1640 medium supplemented with 10% FBS, 100 units/mL penicillin, and 100 μg/mL streptomycin cultured at 37 °C in 5% CO_2_. Media was changed every two days [27]. Cells were washed with 5 mL of DPBS by centrifugation for 5 min at 123× *g*. Cells were resuspended with RPMI 1640 supplemented with 1% FBS and adjusted to 3 × 10^6^ cells/mL. The cell suspension (3 × 10^5^ cells/100 μL) was seeded into each well of a 96-well plate.

THP-1 was coincubated with CFSE-labeled yeast suspensions at MOI of 1 for 1 h at 37 °C. After incubation, plates were kept on ice for 5 min to stop phagocytosis, and wells were washed three times with 200 μL of DPBS by centrifugation at 123× *g*, 4 °C for 5 min. Cocultured cells were incubated on ice with 0.004% trypan blue solution for 15 min to quench extracellular fluorescence, then washed twice with DPBS. Fluorescence was evaluated using flow cytometry (FACSCalibur, Becton Dickinson), and data were analyzed using FlowJo software v10 (FlowJo LLC, Ashland, OR, USA). The fluorescence was determined for each isolate on at least three separate occasions. The Phagocytic Index (PI) was calculated as the product of the percentage of positive cells (% CFSE Positive THP-1 cell) multiplied by the mean of the fluorescence intensity (MFI) of the positive population (MFI of CFSE Positive THP-1 cell). The PI was calculated according to the following formula [28]. PI values were averaged from three separate experiments.

Phagocytosis Index (PI) = % CFSE Positive THP-1 cell × MFI of CFSE Positive THP-1 cell

### 2.9. Amplification and Sequencing of ERG11 Gene

Genomic DNA samples from all isolates were obtained from a previous study [6]. The full-length *ERG11* gene was PCR amplified using primers and amplification conditions detailed in Table S2. The final volume 50 μL PCR reaction mixture contained 0.8 µM of each primer and 25 µL of 2× GoTaq Green master mix (Promega, Madison, WI, USA) containing buffer, nucleotides, and Taq polymerase. All reactions were run on a T100 Thermal Cycler (Bio-Rad Laboratories Inc, Hercules, CA, USA). Amplification products were purified and sequenced in both directions by Macrogen Inc. The entire *ERG11* open reading frame sequence from each *Candida* species was aligned with *C. albicans* ATCC 90028 (GU371851), *C. glabrata* ATCC 90030 (KR998002), *C. tropicalis* ATCC 750 (M23673), and *C. parapsilosis* ATCC 22019 (GQ302972) using Clustal W implemented in the MEGA X software package (v1.0).

### 2.10. Statistical Analysis

Data were analyzed using the statistical software Stata version 11. The Mann–Whitney test was used to calculate statistical differences among isolates of *C. albicans*, *C. parapsilosis*, *C. glabrata*, and *C. tropicalis*. In all tests, a *p* < 0.05 was considered significant.

## 3. Results

### 3.1. Virulence Assessment of Invasive Candida spp. Isolates

We investigated the production of extracellular enzymes, including, phospholipase, protease, and hemolysin in *Candida* spp. that cause BSIs.

**Figure 1:**
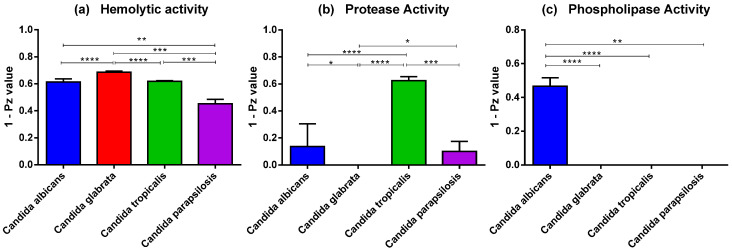
Comparison of the levels of virulence factors of four *Candida* species, (**a**) hemolytic activity, (**b**) protease activity, and (**c**) phospholipase activity. **** *p*  <  0.0001, *** *p*  <  0.001, ** *p*  <  0.01, and * *p*  <  0.05.

All the tested isolates demonstrated potent hemolytic activity on sheep blood SDA plates with Pz values < 0.69 (Table S3). The mean Pz values for the hemolytic activity of *C. albicans*, *C. parapsilosis*, *C. tropicalis*, and *C. glabrata* were 0.39 ± 0.07, 0.55 ± 0.03, 0.38 ± 0.02, and 0.32 ± 0.03, respectively. Figure 1 depicts enzyme activity as a 1-Pz value on the y-axis, with a lower Pz value indicating greater enzyme activity. The average hemolytic activity for *C. glabrata* was significantly higher than that of the other *Candida* spp., indicating *C. glabrata* had the strongest enzyme activity (Figure 1a).

Proteinase activity was found in 57.9% of all isolates. Proteinase production was detected in 100% of the *C. tropicalis* isolates followed by 75% of *C. parapsilosis*, and 50% of *C. albicans* isolates but was not found in any of the *C. glabrata* isolates (Table S3). The mean Pz values for proteinase-positive *C. tropicalis*, *C. parapsilosis*, and *C. albicans* isolates were 0.38 ± 0.03, 0.90 ± 0.10, and 0.86 ± 0.17, respectively, indicating that *C. tropicalis* had a higher proteinase activity than that of *C. albicans* or *C. parapsilosis*. The protease activity of *C. tropicalis* was significantly higher than that of each of the other species (Figure 1b).

Phospholipase activity was detected in 26.3% of the isolates and was present in all *C. albicans* isolates but was undetectable in other *Candida* species (Table S3). The average Pz value in phospholipase-positive *C. albicans* was 0.53 ± 0.05, indicating that *C. albicans* had potent phospholipase activity, which significantly differed from that of the other species (Figure 1c).

### 3.2. Adhesion of Invasive Candida spp. Isolates

**Figure 2:**
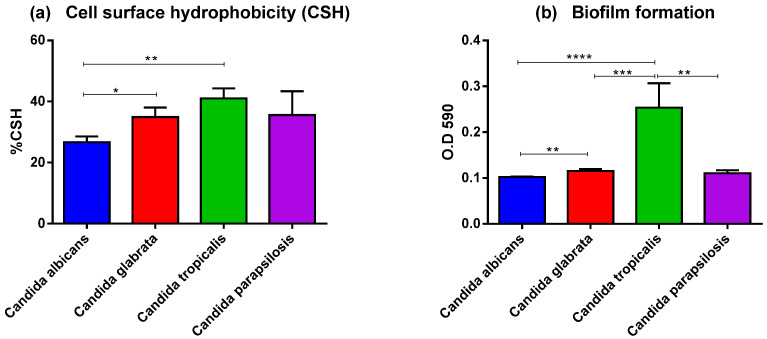
Comparison of cell surface hydrophobicity and biofilm formation for the four *Candida* species, (**a**) Cell surface hydrophobicity (CSH), and (**b**) biofilm formation. **** *p*  <  0.0001, *** *p*  <  0.001, ** *p*  <  0.01, and * *p*  <  0.05.

To evaluate the virulence factors involved in the adhesion process, the CSH and biofilm formation of different *Candida* species were examined. CSH has a considerable influence on interactions between the *Candida* and the host surface. A high CSH is considered to be a virulence factor for numerous fungal species. The determination of CSH on the yeast cell surface is usually related to biofilm formation. CSH was found in varying degrees in all four *Candida* species. The highest average CSH value was found in *C. tropicalis* (41.0 ± 12.20%), followed by *C. parapsilosis* (35.5 ± 15.66%), *C. glabrata* (34.9 ± 9.75%), and *C. albicans* (26.7 ± 5.75%). The %CSH significantly differed between *C. albicans* and *C. tropicalis* as well as between *C. albicans* and *C. glabrata* (Figure 2a).

The biomass of the *Candida* biofilm was quantified at 48 h with a crystal violet assay. *C. tropicalis* isolates produced greater total biofilm formation than that in *C. albicans*, *C. glabrata,* or *C. parapsilosis* (average OD at 590 nm: 0.25 ± 0.2, 0.1 ± 0.01, 0.12 ± 0.01, and 0.11 ± 0.02, respectively), When each *Candida* species was compared, *C. tropicalis* produced significantly more biofilm than that in the other species (Figure 2b).

### 3.3. Ability to Invade and Damage Epithelial Cells

**Figure 3:**
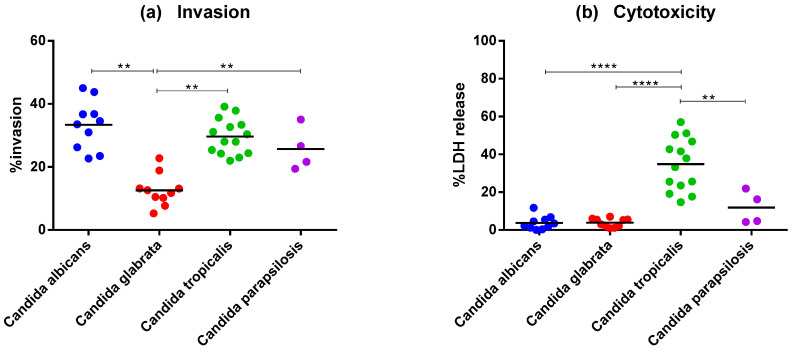
Comparison of *Candida* invasion and epithelia host cell damage among different species. (**a**) A459 cells were cocultured with each *Candida* species at an MOI of 5 for 4 h. The percentage of invading yeast cells was determined. (**b**) A459 cells were cocultured with each *Candida* species at an MOI of 5 for 18 h. Cell supernatants were analyzed for LDH presence. **** *p*  <  0.0001, and ** *p*  <  0.01.

We compared the invasion potential of each *Candida* species using the A459 epithelial cell monolayer infection model. *C. albicans* (33.37%) had the highest percentage of invasion after 4 h of infection, followed by *C. tropicalis* (29.64%), *C. parapsilosis* (25.68%), and *C. glabrata* (12.60%). *C. glabrata* demonstrated a much lower invasion rate compared with that of other *Candida* species. The degree of invasion between *C. albicans*, *C. tropicalis*, or *C. parapsilosis* was not significantly different (Figure 3a).

The cell damage caused by each *Candida* species was tested in A549 monolayer culture by measuring the amount of LDH released in culture supernatants during 18 h of infection. The amount of LDH released due to *C. tropicalis* (34.8%) was significantly higher than that released due to another *Candida* spp. The amount of LDH released due to *C. albicans* (3.77%), *C. glabrata* (3.85%), or *C. parapsilosis* (11.75%) (Figure 3b) was not significantly different among these species.

### 3.4. Morphologic Transition in Candida spp. Isolates

**Figure 4:**
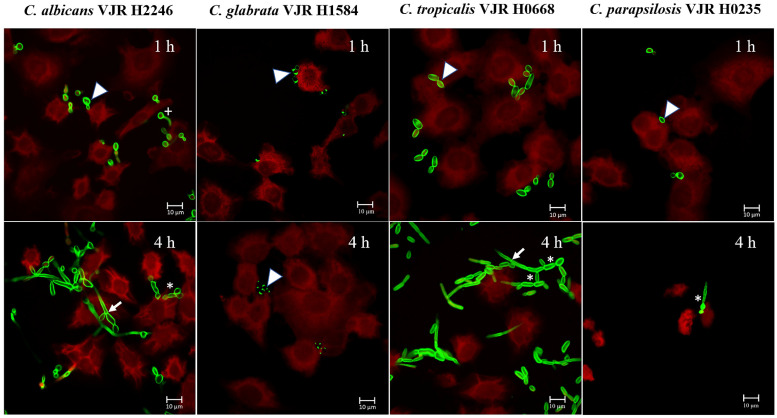
Confocal microscopy images of A459 epithelial cells and *Candida* spp. interaction at 1 and 4 h. Calcofluor white-stained *Candida* cell walls (green) allow visualization of morphology of yeast cell/budding yeast (arrowhead), true hyphae (arrow), germ tube (+), and pseudohyphae (*). Phalloidin-conjugated Alexa Fluor 647 was used to label F-actin to show the cytoskeleton morphology within A549 cells.

Confocal microscopy of A549 epithelial cells cocultured with each *Candida* species was used to assess the morphological changes of different *Candida* spp., after 1 and 4 h of co-culturing. After the first hour of infection, all *Candida* species adhered to the epithelial cell surface as budding yeast cells, although only *C. albicans* had formed germ tubes. At 4 h after infection, both *C. albicans* and *C. tropicalis* exhibited pseudohyphae and hyphae that pierced the epithelial host cell, whereas *C. glabrata* was only present as the yeast form on the epithelial surface, and *C. parapsilosis* had changed morphologically into pseudohyphae (Figure 4).

### 3.5. Phagocytosis Assay with Candida spp. Isolates

**Figure 5:**
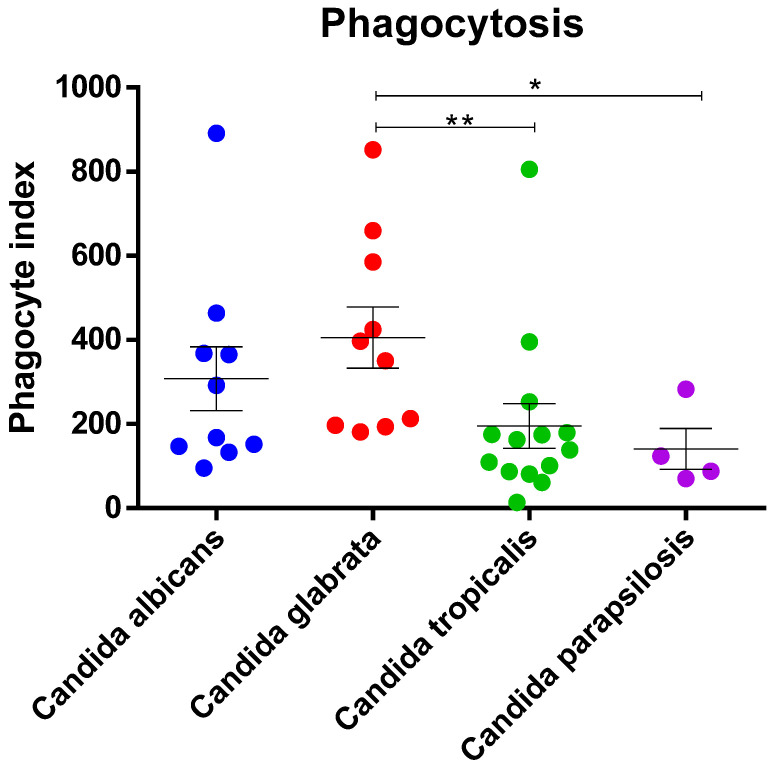
Comparison of phagocytic index of different *Candida* species by THP-1 cells. THP-1 cells were cocultured with each *Candida* species at an MOI of 1 for 1 h. Phagocytosis was determined via flow cytometry, and the results are expressed as the phagocytic index. ** *p*  <  0.01, * *p*  <  0.05, and not significant *p*  ≥  0.05.

To compare the level of immune cell phagocytosis of different *Candida* species, human monocytes (THP-1) were infected with live *Candida* spp. at an MOI of 1, and the level of phagocytosis was measured via flow cytometry. *C. glabrata* had the highest phagocytic index followed by *C. albicans, C. tropicalis*, and *C. parapsilosis*. *C. glabrata* was significantly more phagocytosed by THP-1 cells than *C. tropicalis* and *C. parapsilosis* were (Figure 5).

### 3.6. Mutation in ERG11 Gene

The amino acid substitution in the ERG11 protein sequence contributes to azole resistance in clinical isolates. The complete sequence of the *ERG11* gene in each of the 38 *Candida* isolates was amplified and sequenced to identify amino acid polymorphisms. The susceptibility profiles of the isolates from our previous study are shown in Table S1. All *C. albicans* isolates were susceptible to all azole antifungal drugs tested, although 50% of *C. albicans* isolates had *ERG11* substitutions. Four distinct *ERG11* substitutions in *C. albicans* (D116E, D153E, K342R, and E226D) were identified. For *C. tropicalis*, four (28.6%) of the 14 *C. tropicalis* isolates were resistant to fluconazole, while ten were susceptible. Two *ERG11* substitutions (Y132F and S154F) were commonly found in fluconazole-resistant *C. tropicalis*. No *ERG11* mutations were identified in isolates of *C. parapsilosis* or *C. glabrata* (Table S3).

## 4. Discussion

*C*. *albicans* is the most common cause of candidemia worldwide, and NAC spp. are becoming more common, particularly in the Asia–Pacific region [6,29,30]. Insufficient information exists on the virulence properties of *Candida* spp. that cause candidemia in Thailand; therefore, we characterized the virulence factors of *Candida* spp. isolated from patients with BSIs as well as the presence of *ERG11* mutations that contribute to azole-resistance. We found *ERG11* mutations in *C. albicans* and *C. tropicalis* isolates but not in *C. glabrata* or *C. parapsilosis*. Thus, the *ERG11* mutation may not be a major resistant mechanism in *C. glabrata* and *C. parapsilosis* isolates in Thailand. In *C. albicans*, four missense mutations (D116E, D153E, K342R, and E226D) were found in fluconazole-susceptible isolates. Although a previous study showed that the majority of the fluconazole-susceptible isolates had only one *ERG11* missense mutation [31], we discovered the coexistence of D116E and D153E in a fluconazole-susceptible *C. albicans* isolate; however, the coexistence of D116E and D153E has been demonstrated in azole-resistant isolates [32]. The *ERG11* mutation in *C. albicans* did not cause azole resistance in our isolate. For *C. tropicalis*, 28.5% of the isolates contained two distinct amino acid alterations (Y132F and S154F), which commonly coexist and were detected in all isolates resistant to fluconazole. This is a similar result to that in previous studies where the Y132F and S154F mutations in ERG11 were strongly associated with azole-resistant phenotype and suggested that these mutations could be potential predictive markers of azole resistance [33,34]. The Y132F mutation is responsible for the loss of the normal hydrogen bonding between tyrosine and heme. This alteration affects drug binding because heme is a binding cofactor of azole for *ERG11* [35,36,37]. Substitutions of both Y132F and S154F prevented the development of Pi-Pi and Pi-cation interactions between the cofactor and the ligand molecules, which lowers the overall binding energy of the altered docked complex [35].

As a pathogen, *Candida* spp. must first attach to various host cells. We evaluated the virulence factors involved in the adhesion process, such as CSH and biofilm formation in 38 strains of *Candida* species isolated from patients with a BSI. CSH is an important virulence factor that contributes to fungal pathogenicity and is essential for cell adhesion to human epithelial cells as well as to catheters or medical devices implanted in patients [38,39]. Fungal adhesion to surfaces and subsequent biofilm formation is critical because this can lead to antifungal drug resistance. A high CSH is a common feature of disease-causing yeast isolates [40,41]. *C. tropicalis* had the highest hydrophobicity values in the current study, followed by those of *C. parapsilosis*, *C. glabrata*, and *C. albicans*. Consistent with previous research, NAC, as ordered by *C. tropicalis*, *C. parapsilosis*, and *C. glabrata*, have a greater CSH value than that of *C. albicans* [42]. Biofilm formation is also important in *Candida* pathogenesis because this protects pathogenic yeast from host immune cells and causes resistance to antifungal treatment. Additionally, biofilm development facilitates *Candida* adhering to indwelling medical equipment such as cardiac devices, artificial joints, and vascular catheters [43]. Our results revealed that *C. tropicalis* had the highest biomass production followed by *C. parapsilosis*, *C. glabrata*, and *C. albicans*, respectively. This result agrees with previous studies where NAC strains could produce more biofilm than *C. albicans* could [42,44,45]. Biofilm formation in this study was related to the presence of CSH.

Following adherence, the pathogen must invade the cells. The invasion and damage to the epithelium are mostly regarded as pathogenic properties [46]. To enable penetration of the epithelial cell barrier, *Candida* secretes a series of hydrolytic enzymes such as hemolysin, protease, and phospholipase that digest the host cell membrane and defend against the immune response of the host [47]. Hemolysin production is an important virulence factor for *Candida* because hemolysin lyses red blood cells to obtain iron sourced from hemoglobin, supporting hyphal penetration and yeast dissemination in the host [48,49]. In our study, all the clinical isolates were able to produce hemolysins. These findings corroborate those in a previous study that evaluated the hemolysin activity of 70 clinical isolates from patients with BSIs [50]. Phospholipases hydrolyze phospholipids as substrates, causing host cell membranes to rupture, and the secretion of phospholipases from *Candida* species is involved in tissue invasion [51]. *C. albicans* has strong phospholipase activity, but NAC spp. can exhibit lower or negative phospholipase production [15,52]. Corroborating these studies, all *C. albicans* isolates in this study produced strong phospholipase activity, whereas none of the NAC spp. exhibited this activity. However, a previous study identified that the majority of phospholipase producers were NAC strains, with *C. tropicalis* and *C. parapsilosis* exhibiting marked phospholipase activity [53]. Proteases can degrade host epithelial and mucosal barrier proteins, including albumin, collagen, and mucin, aiding *Candida* in resisting host immune system attacks by degrading antibodies, complement factors, and cytokines [17,54]. *Candida*-secreted aspartyl proteases (SAPs) can enhance hypha formation, epithelial cell damage, and the maintenance of biomass as well as increase yeast invasiveness in in vivo models [55,56]. We found that the detection rate of proteinase production in NAC isolates from blood (60.7%) was higher than in *C. albicans* (50%), which contradicts previous studies where the proteinase activity was present at a lower rate in NAC isolates than that in *C. albicans* [57,58]. Among the NAC isolates in this study, 100% of *C. tropicalis* and 25% of *C. parapsilosis* isolates were protease producers. Individual isolates of *C. tropicalis* in our investigation showed strong levels of proteinase activity compared with that in other *Candida* spp. isolates. No strains of *C. glabrata* were protease-positive strains. Our results do not agree with a previous study that reported that 80% of *C. glabrata* isolates from blood were protease producers [57]. The differences in hydrolytic enzyme patterns may vary depending on the strains present in geographical regions and the host populations.

After adherence and hydrolytic enzyme secretion, *Candida* spp. can invade and damage the host epithelial cells. *Candida* invades epithelial cells via two distinct mechanisms: inducing endocytosis and active penetration [59]. The induction of endocytosis contributes to an early point of the invasion process, whereas active penetration is the main epithelial invasion mechanism [60]. The morphologic transition switching between the yeast and hyphal forms represents a virulence factor that facilitates the active invasion process. Our findings support previous research that *C. albicans* and *C. tropicalis* can form hyphal filamentous structures that penetrate epithelial cells. In this study, *C. parapsilopsis* strains were unable to form true hyphae but did undergo a morphologic shift to the pseudohyphal form. *C. glabrata* cannot form elongated pseudohyphae or true hyphae structures, which is consistent with previous research [61,62]. We demonstrated that the formation of true hyphal filamentous or pseudohyphae in *Candida* isolates such as *C. albicans* and *C. tropicalis* correlates with their invasion ability in a host epithelium model where *C. albicans* had the highest level of invasion, followed by *C. tropicalis*, *C. parapsilosis*, and *C. glabrata*. Using the release of the intracellular marker enzyme LDH from the epithelial cells into the culture media, we evaluated the damage to epithelial cells caused by different *Candida* species. *C. tropicalis* infection significantly increased epithelial cell injury within 18 h of infection in comparison with the effect of other *Candida* species.

Monocytes are blood-borne phagocytic cells that encounter and eliminate microbes and yeast cells. We demonstrated that monocytes could more efficiently phagocytose *C. glabrata*, as indicated by a higher phagocyte index than that of other *Candida* spp.; *C. tropicalis* and *C. parapsilosis* were more resistant to phagocytosis, with the lowest phagocyte index. The differences in phagocyte-uptake efficiency of *Candida* spp. corresponded well with *C. glabrata* being less virulent than other species in this study. The difference in phagocyte recognition of the four *Candida* spp. depends on the cell wall composition of pathogen-associated molecular patterns, including the presence of mannans, mannoproteins, chitin, and β-glucans. The cell wall of *C. glabrata* contains 50% more mannose than *C. albicans* [63], and *C. glabrata* is more likely to be more easily recognized by phagocyte mannose-specific receptors (mannose receptor, TLR4, dectin-2, and galectine-3) [64]. *C. albicans* modifies the glycan component on the cell surface and does not expose β-glucans in the true hyphae state, which prevents activation of the dectin-1 receptor involved in phagocytosis [65,66,67]. However, this study only demonstrated the level of uptake of different *Candida* species into the phagocytic cell but not killing activity. Further study is needed to elucidate the rate of host cell killing and intracellular survival mechanisms among *Candida* species.

With the increasing prevalence of *C. tropicalis* infections in our country and the occurrence of drug resistance, anti-virulence therapy in combination with antifungal drug treatment may be beneficial in the fight against *C. tropicalis*. Considering the proteases involved in biofilm formation, hyphal production, and epithelial cell damage, this study observed a statistically significant increase in the production of proteases, biofilm formation, and epithelial cell damage among *C. tropicalis* isolates compared to other species. Among these virulent features, proteases may play a critical role in the pathogenesis of *C. tropicalis* infection in our study. Biofilms formed by *C. tropicalis* are resistant to many antifungal agents, which makes them very difficult to treat. Therefore, strategies for overcoming this problem may include the use of protease inhibitors in combination with antifungal drugs. Aspartyl protease inhibitors can reduce *Candida*-induced tissue damage, proliferation, and virulence in vivo as well as reduce *Candida* adherence to the materials commonly used in medical devices [68,69,70,71,72]. Previous reports showed that treatment with aspartyl protease inhibitors reduced the occurrence of oral candidiasis in immunocompromised patients [73,74]. Optimistically, information from the current study will be useful for understanding the pathogenicity process of *Candida* spp. in Thailand and assisting physicians in making treatment decisions to target virulence factors in combination with antifungal drugs against *Candida* infections.

## 5. Conclusions

This study provides vital information regarding the pathogenicity of *Candida* spp. causing BSIs in Thailand. The current study demonstrates that *Candida* spp. in Thailand display distinct characteristics of virulence factor expression. *C. tropicalis* exhibits biofilm, hydrophobicity, protease activity, host cell damage, and true hyphae formation, whereas *C. albicans* has the highest epithelial cell invasion rate, phospholipase activity, and true hyphae formation. *C. glabrata* produces the most hemolysis activity, whereas *C. parapsilosis* produces pseudohyphae and is resistant to monocyte phagocytosis. Knowledge of these virulence factors will be crucial for understanding the pathogenicity of *Candida* species and helping explore treatment. The findings in this study raise awareness concerning the deleterious effects of *C. tropicalis*, the most common cause of BSI in Thailand, which harbor several virulence features such as dimorphism, biofilm formation, high hydrophobicity, and phagocytosis resistance.

## Data Availability

Not applicable.

## Supplementary Materials

The following supporting information can be downloaded at: https://www.mdpi.com/article/10.3390/jof9030353/s1, Table S1: Strain information of 38 clinical isolates of *C. albicans*, *C. glabrata*, *C. tropicalis*, and *C. parapsilosis* used in this study; Table S2: List of primers and PCR conditions used for full-length ERG11 gene amplification and sequencing; Table S3: Virulence factors and ERG11 mutations of *Candida* spp. blood isolates of patients with candidemia admitted at tertiary hospitals in Bangkok, Thailand.
