# Supplementary material for: Characterization of Virulence Factors in Candida Species Causing Candidemia in a Tertiary Care Hospital in Bangkok, Thailand
Source: J Fungi (Basel). 2023 Mar 14;9(3):353. doi: 10.3390/jof9030353 (PMC10059995; doi:10.3390/jof9030353)
Supplement: Supplementary file 1 [file jof-09-00353-s001.zip › jof-2225231-supplementary.pdf]

Journal of *Fungi*

MDPI

Table S1. Strain information of 38 clinical isolates of *C. albicans*, *C. glabrata*, *C. tropicalis*, and *C. parapsilosis* used in this study [6].

Species	Strains	Susceptibility profiles		
		Fluconazole	Itraconazole	Voriconazole
C. albicans	VJR H0290	S	NA	S
	VJR H0432	S	NA	S
	VJR H0969	S	NA	S
	VJR H1000	S	NA	S
	VJR H1651	S	NA	S
	VJR H1976	S	NA	S
	VJR H2010	S	NA	S
	VJR H2048	S	NA	S
	VJR H2090	S	NA	S
	VJR H2246	S	NA	S
C. glabrata	VJR H0454	SDD	WT	WT
	VJR H0789	SDD	WT	non-WT
	VJR H1584	SDD	WT	non-WT
	VJR H1611	SDD	WT	non-WT
	VJR H1642	R	WT	non-WT
	VJR H1688	SDD	WT	non-WT
	VJR H1783	SDD	WT	non-WT
	VJR H2208	SDD	WT	non-WT
	VJR H2413	R	WT	non-WT
	VJR H2415	SDD	WT	non-WT
C. tropicalis	VJR H0040	R	non-WT	R
	VJR H0315	I	non-WT	I
	VJR H0593	R	non-WT	R
	VJR H0668	S	WT	I
	VJR H0675	S	WT	S
	VJR H1039	S	non-WT	S
	VJR H1322	S	WT	S
	VJR H1343	R	non-WT	R
	VJR H1538	S	WT	S
	VJR H1541	S	WT	S
	VJR H1699	S	WT	S
	VJR H1775	S	WT	S
	VJR H2316	S	WT	S
	VJR H2467	R	non-WT	R
C. parapsilosis	VJR H0084	S	NA	S
	VJR H0143	I	NA	S
	VJR H0235	I	NA	S
	VJR H1311	S	NA	S

S, susceptible; I, intermediate; R, resistant; SDD, susceptible dose dependent; WT, wild type; NA, not applicable. WT defined by an epidemiological cutoff value (ECV) that describes isolates with no resistance mechanisms to antimicrobial agent being evaluated. non-WT defined by an ECV that describes isolates with the present of resistance mechanisms to antimicrobial agent being evaluated.

Table S2. List of primers and PCR conditions used for full-length ERG11 gene amplification and sequencing.

Primer name (Reference)	Primer Sequence (5′ to 3′)	Product Size (bp.)	Application	PCR Condition
C. albicans				
caERG11_AmpF (this study)	ATAGACAAAGAAAGGGAATTC	1765	PCR Sequencing	95°C for 5 min, 30 cycles of 95°C for 1 min, 53°C for 1 min, 72°C for 1.30 min, followed by 72°C for 5 min
caERG11 Amp R (this study)	ATGTGTATATATGTTAATCCAAC		PCR Sequencing	
caERG11 Seq R (this study)	TTTGGATCAATATCACCACG	-	Sequencing	-
caERG11 Seq F (this study)	GGAGACGTGATGCTGCTCAA	-	Sequencing	-
C. glabrata				
cgERG11 Amp F (this study)	GTTCTCATTGTCTATTTCGATACT	1753	PCR Sequencing	95°C for 5 min, 30 cycles of 95°C for 1 min, 53°C for 1 min, 72°C for 1.30 min, followed by
cgERG11 Amp R (this study)	TATATCCCGTATACGAGCC		PCR Sequencing	

Primer name (Reference)	Primer Sequence (5′ to 3′)	Product Size (bp.)	Application	PCR Condition
				72°C for 5 min
cgERG11 Seq F (this study)	GCCTCTAGAACACTACAGAA	-	Sequencing	-
cgERG11 Seq R (this study)	TTCATCAATCAAGTCACGGT	-	Sequencing	-
C. parapsilosis				
cpERG11 Amp F (this study)	TGCTACTAACTTTCCCTACC	1684	PCR Sequencing	95°C for 5 min, 30 cycles of 95°C for 1 min, 53°C for 1 min, 72°C for 1.30 min, followed by 72°C for 5 min
cpERG11 Amp R (this study)	GCATTAAACCCCATTGATTC		PCR Sequencing	
cpERG11 Seq F (this study)	GGATTCACCCCAATCAATTT	-	Sequencing	-
cpERG11 Seq R (this study)	TTCTAGCAATCTCTGTCATATAC	-	Sequencing	-
C. tropicalis				
ctERG11 Amp F1 [12]	TGAAGAATATCCCACAGGCT	1057	PCR Sequencing	95°C for 5 min, 30 cycles of 95°C for 1 min, 56°C for 1 min, 72°C for 1 min, followed by 72°C for 5 min
ctERG11 Amp R1 (this study)	TTGACCACCCATCAAAACACC		PCR Sequencing	
ctERG11AmpF2 (this study)	GTTTCACCCCAATCAA	982	PCR Sequencing	95°C for 5 min, 30 cycles of 95°C for 1 min, 45°C for 1 min, 72°C for 1 min, followed by 72°C for 5 min
ctERG11AmpR2 (this study)	TGTATACTGTATTAAAGGC		PCR Sequencing	

Table S3. Virulence factors and ERG11 mutations of *Candida* spp. blood isolates of patients with candidemia admitted at tertiary hospitals in Bangkok, Thailand.

Strains	Hemolysis Zone (Pz)	Phospholipase Zone (Pz)	Proteinase Zone (Pz)	Biofilm Formation (OD570)	% CSH	ERG11 Mutation
C. albicans H0290	0.35±0.01	0.49±0.08	0.74±0.02	0.10±0.03	21.78±13.74	D116E, D153E[#]
C. albicans H0432	0.59±0.25	0.60±0.10	1.00±0.00	0.10±0.01	22.64±6.32	K342R
C. albicans H0969	0.35±0.05	0.57±0.01	0.58±0.10	0.11±0.00	30.08±18.04	D153E
C. albicans H1000	0.38±0.05	0.55±0.04	1.00±0.00	0.10±0.01	31.46±10.14	-
C. albicans H1651	0.35±0.01	0.44±0.07	1.00±0.00	0.10±0.00	20.79±14.84	E226D
C. albicans H1976	0.36±0.01	0.51±0.03	0.88±0.14	0.10±0.01	31.65±5.05	-
C. albicans H2010	0.36±0.02	0.54±0.03	0.60±0.08	0.11±0.01	38.16±8.94	-
C. albicans H2048	0.35±0.05	0.58±0.04	1.00±0.00	0.10±0.00	25.21±4.18	D153E
C. albicans H2090	0.36±0.02	0.56±0.03	1.00±0.00	0.10±0.00	23.34±3.17	-
C. albicans H2246	0.41±0.06	0.49±0.03	0.84±0.19	0.10±0.00	22.37±4.63	-
C. glabrata H0454	0.31±0.03	1.00±0.00	1.00±0.00	0.15±0.01	60.32±8.01	-
C. glabrata H0789	0.33±0.04	1.00±0.00	1.00±0.00	0.11±0.00	34.75±9.48	-
C. glabrata H1584	0.28±0.02	1.00±0.00	1.00±0.00	0.12±0.00	32.17±9.15	-
C. glabrata H1611	0.28±0.01	1.00±0.00	1.00±0.00	0.12±0.00	35.93±9.84	-
C. glabrata H1642*	0.29±0.04	1.00±0.00	1.00±0.00	0.11±0.00	29.25±9.13	-
C. glabrata H1688	0.31±0.05	1.00±0.00	1.00±0.00	0.10±0.00	39.90±11.35	-
C. glabrata H1783	0.32±0.01	1.00±0.00	1.00±0.00	0.10±0.00	25.87±10.27	-
C. glabrata H2208	0.35±0.02	1.00±0.00	1.00±0.00	0.11±0.01	31.73±9.62	-
C. glabrata H2413*	0.37±0.01	1.00±0.00	1.00±0.00	0.12±0.00	29.34±10.10	-
C. glabrata H2415	0.31±0.02	1.00±0.00	1.00±0.00	0.11±0.00	30.17±4.98	-
C. parapsilosis H0084	0.61±0.01	1.00±0.00	1.00±0.00	0.13±0.05	58.99±3.77	-
C. parapsilosis H0143	0.59±0.07	1.00±0.00	0.83±0.19	0.10±0.02	26.92±2.12	-
C. parapsilosis H0235	0.47±0.04	1.00±0.00	0.91±0.19	0.10±0.02	29.23±3.16	-
C. parapsilosis H1311	0.52±0.05	1.00±0.00	0.86±0.16	0.11±0.01	27.07±5.65	-
C. tropicalis H0040*	0.38±0.02	1.00±0.00	0.37±0.02	0.19±0.05	36.50±6.51	Y132F, S154F
C. tropicalis H0315	0.37±0.01	1.00±0.00	0.38±0.00	0.57±0.16	47.62±13.92	-
C. tropicalis H0593*	0.39±0.03	1.00±0.00	0.38±0.03	0.19±0.01	33.80±11.33	Y132F, S154F
C. tropicalis H0668	0.38±0.02	1.00±0.00	0.36±0.03	0.25±0.04	26.95±6.81	-
C. tropicalis H0675	0.38±0.04	1.00±0.00	0.33±0.06	0.83±0.55	51.28±7.54	-
C. tropicalis H1039	0.40±0.03	1.00±0.00	0.37±0.02	0.11±0.00	22.24±4.28	-
C. tropicalis H1322	0.37±0.06	1.00±0.00	0.36±0.01	0.20±0.01	60.62±12.24	-
C. tropicalis H1343*	0.39±0.01	1.00±0.00	0.38±0.00	0.21±0.05	55.62±4.82	Y132F, S154F
C. tropicalis H1538	0.39±0.04	1.00±0.00	0.36±0.02	0.18±0.01	30.33±11.70	-

Strains	Hemolysis Zone (Pz)	Phospholipase Zone (Pz)	Proteinase Zone (Pz)	Biofilm Formation (OD570)	% CSH	ERG11 Mutation
C. tropicalis H1541	0.40±0.02	1.00±0.00	0.39±0.03	0.13±0.01	56.94±2.51	-
C. tropicalis H1699	0.38±0.03	1.00±0.00	0.37±0.02	0.15±0.00	42.39±3.48	-
C. tropicalis H1775	0.34±0.02	1.00±0.00	0.47±0.02	0.15±0.05	46.40±13.36	-
C. tropicalis H2316	0.40±0.04	1.00±0.00	0.38±0.04	0.18±0.02	30.58±13.79	-
C. tropicalis H2467*	0.37±0.03	1.00±0.00	0.37±0.02	0.21±0.02	33.31±7.08	Y132F, S154F

Data were represented as mean ± SD. * denoted the fluconazole-resistant strain. # denoted the heterozygous mutation in ERG11. Amino acid abbreviations: D, (aspartic acid, Asp); E, (glutamic acid, Glu); F, (phenylalanine, Phe); K, (lysine, Lys); R, (arginine, Arg); V, (valine, Val); Y, (tyrosine, Tyr).
